# Asymmetric, amphiphilic RGD conjugated phthalocyanine for targeted photodynamic therapy of triple negative breast cancer

**DOI:** 10.1038/s41392-022-00906-2

**Published:** 2022-02-28

**Authors:** Rui Li, Yiming Zhou, Yijia Liu, Xingpeng Jiang, Wenlong Zeng, Zhuoran Gong, Gang Zheng, Desheng Sun, Zhifei Dai

**Affiliations:** 1 Department of Biomedical Engineering, College of Future Technology, National Biomedical Imaging Center, Peking University, Beijing, 100871 China; 2 Princess Margaret Cancer Centre, University Health Network, 101 College Street, PMCRT 5-354, Toronto, Ontario M5G 1L7 Canada; 3 Department of Medical Biophysics, University of Toronto, Toronto, Ontario M5G 1L7 Canada; 4 Department of Ultrasonic Imaging, Peking University Shenzhen Hospital, Shenzhen, China

**Keywords:** Breast cancer, Drug development

## Abstract

Targeted photodynamic therapy (TPDT) is considered superior to conventional photodynamic therapy due to the enhanced uptake of photosensitizers by tumor cells. In this paper, an amphiphilic and asymmetric cyclo-Arg-Gly-Asp-d-Tyr-Lys(cRGDyK)-conjugated silicon phthalocyanine (RSP) was synthesized by covalently attaching the tripeptide Arg-Gly-Asp (RGD) to silicone phthalocyanine in the axial direction for TPDT of triple-negative breast cancer (TNBC). RSP was characterized by spectroscopy as a monomer in physiological buffer. Meanwhile, the modification of RSP with RGD led to a high accumulation of the photosensitizer in TNBC cells overexpressing ανβ3 integrin receptors which can bind RGD, greatly reducing the risk of phototoxicity. In vitro photodynamic experiments showed that the IC50 of RSP was 295.96 nM in the 4T1 cell line, which caused significant apoptosis of the tumor cells. The tumor inhibition rate of RSP on the orthotopic murine TNBC achieved 74%, while the untargeted photosensitizer exhibited no obvious tumor inhibition. Overall, such novel targeted silicon phthalocyanine has good potential for clinical translation due to its simple synthesis route, strong targeting, and high therapeutic efficacy for TPDT treatment of TNBC.

## Introduction

Photodynamic therapy (PDT), a minimally invasive and high-efficient treatment, is less likely to develop drug resistance,^1,2^ and it has been applied to the treatment of TNBC.^3–6^ Targeted photodynamic therapy (TPDT) was developed to improve the delivery of photosensitizer (PS), the most central element of PDT,^7^ to cancerous tissues while increasing the specificity and efficiency of PDT.^8^ On the one hand, TPDT reduces phototoxicity to the normal cells and enhances damage to the tumors^9^; on the other hand, the injection dose of PS is substantially reduced,^10^ and TPDT shows greater advantages for tumors that develop resistance.^11^ Therefore, TPDT is a promising option for the precise photodynamic therapy of cancer.

The implementation of TPDT is mainly dependent on the targeted modification of PS.^12^ The design of clinically available target PS requires not only single component, water solubility, and high singlet oxygen (^1^O_2_) yield but also good targeting ability and excellent cell affinity.^13,14^ Phthalocyanine, a class of photosensitizers with strong absorption in the near-infrared region, possesses the strong potential for clinical applications.^15–17^ In the past decade, targeting modifications of phthalocyanines have been mainly divided into “passive targeting” and “active targeting”, which is designed based on molecular recognition of drug delivery.^18–21^ Due to the heterogeneity of tumors, the permeability of blood vessels within the same tumor may be different,^22^ and relying only on passive delivery systems will inevitably encounter its inherent limitations and result in limited therapeutic efficacy.^23^ Therefore, targeted modification of phthalocyanine molecules to give the molecule an active targeting function is a more promising approach for precise tumor targeting.

Triple-negative breast cancer (TNBC) is a highly aggressive subclass of breast cancer. Targeted therapy of the TNBC remains a major challenge due to the lack of the effective targets and drug resistance. Based on the intrinsic characteristics of TNBC, preclinical trials of various targeted therapy have been performed for TNBC.^24,25^ Integrin α_ν_β_3_ (a transmembrane glycoprotein receptor heterodimer overexpressed on the surface of tumor vessels and a variety of tumor cells) is commonly overexpressed by many malignancies, including TNBC, which is the basis for TNBC-targeted therapy using α_ν_β_3_-specific ligands.^26^ Previous studies showed that RGD is a ligand for α_ν_β_3_-integrins and can readily bind to α_ν_β_3_ integrin receptors with high affinity and specificity.^27,28^ Recently, various RGD-functionalized small molecule drugs and nano-systems have been developed for the targeted therapy of TNBC.^29–31^ However, there is still no facile ways to prepare the targeted phthalocyanine for the TPDT due to the complexity of the synthesis.

**Scheme 1:**
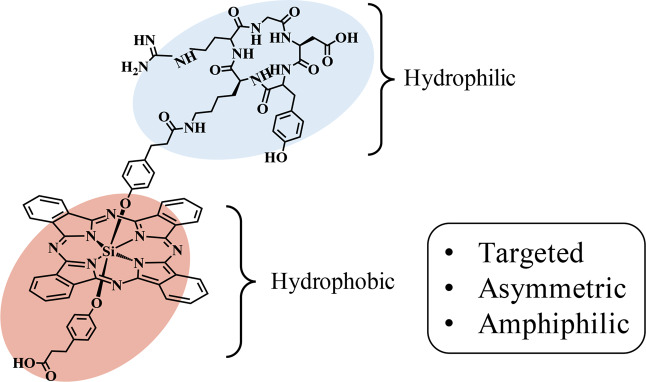
Schematic illustration of amphiphilic and asymmetric cyclo-Arg-Gly-Asp-d-Tyr-Lys(cRGDyK)-conjugated silicon phthalocyanine (RSP)

In this study, we designed and synthesized a novel amphiphilic and asymmetric cyclo-Arg-Gly-Asp-d-Tyr-Lys(cRGDyK)-conjugated silicon phthalocyanine (RSP) (Scheme 1). The cRGDyK moiety enhanced both the targeting ability and the hydrophilicity of RSP, while the whole molecule also exhibited amphiphilicity because of the hydrophobicity of the large pericyclic ring of phthalocyanine. This amphiphilic molecule shows easier uptake by cells than the highly hydrophilic drugs. Molecular simulations and spectroscopic characterizations showed that the axial modification of phthalocyanine effectively avoided the formation of H-aggregates and J-aggregates, and had good singlet oxygen yield and photostability. By RGD targeting, the fluorescence intensity of RSP in orthotopic mouse tumors was 4-folds higher than that of the control. We found that RSP has a half-life of about 45 min in circulation and is metabolized through the liver and kidneys. Furthermore, RSP showed superior tumor targeting than non-targeted silicone phthalocyanine and could inhibit tumor growth more effectively with a tumor inhibition rate of 74%. In summary, the non-aggregated targeted silicon phthalocyanine can be synthesized inexpensively on a large scale, represents a promising next-generation photodynamic therapeutic modality.

## Results

### Molecule design considerations and simulations

**Fig. 1:**
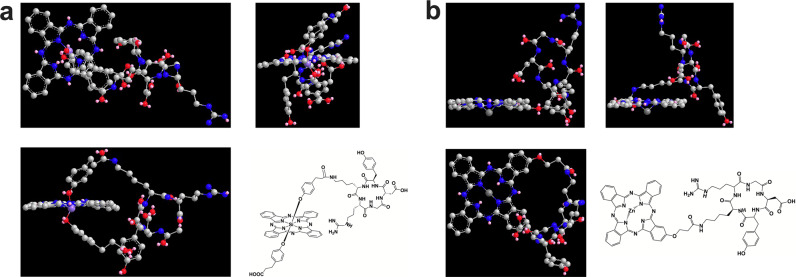
The geometric structure of RSP simulated. (**a**) and the zinc phthalocyanine modified with cRGDyK on the side simulated (**b**) with the MM2 force field to obtain the lowest energy state conformation diagram

The basic principle for the design of targeted photosensitizers is to ensure the in vivo tumor targeting and cellular affinity while effectively reducing photosensitizer aggregation. We axially modified one end of silica phthalocyanine with cRGDyK to obtain amphiphilic, asymmetric targeting photosensitizers. In order to examine the rationality and feasibility of the molecular design as much as possible, we simulated the lowest energy conformation of RSP (Fig. 1a). As a control, the molecular state of zinc phthalocyanine modified with cRGDyK on the side was also simulated (Fig. 1b). From the results, it can be seen that due to the existence of the two benzene rings modified in the axial direction, the macrocyclic planes of the phthalocyanine molecules of RSP are well separated. It can also be seen that the recognition site of the cRGDyK molecule is well exposed, which ensures its targeting ability. However, the molecular plane macrocycles of zinc phthalocyanine are completely exposed which leads to the formation of H-type aggregates, reducing the yield of fluorescence and singlet oxygen. The results of a molecular simulation further confirm that the molecules designed in this paper are less likely to aggregate in aqueous solutions, and therefore have stronger applicability than non-axially modified zinc phthalocyanine.

### Synthesis and Characterization

The synthetic route of RSP was shown in Supplementary Fig. 1. First, we synthesized silicon dichloride phthalocyanine using easily available 1,3-diiminoisoindolines and silicon tetrachloride as raw materials.^32^ Then, the silicon dichloride phthalocyanine was axially substituted with p-hydroxyphenyl propionic acid under alkaline conditions to obtain a silicone phthalocyanine with two free carboxyl groups (SC, Supplementary Fig. 1, compound **2**). Finally, the RGD targeting group was conjugated to the phthalocyanine. In order to avoid self-cyclization of the cRGDyK molecule and the formation of modified molecules at both ends, activation of one of the carboxyl groups of SC was first performed with the activating ester. After the activation of the carboxyl group, a simple amide condensation reaction at room temperature by strictly controlling the ratio of cRGDyK and SC (1:3) was carried out to initially obtain cRGDyK-substituted silicon phthalocyanines (RSP, Supplementary Fig. 1, compound **3**). The structures of SC and RSP were verified by NMR and MS (Supplementary Figs. 2–4).

**Fig. 2:**
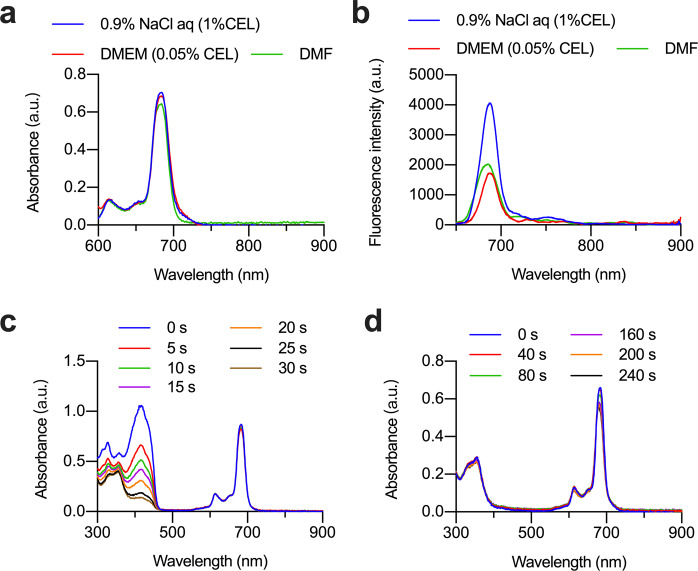
Photophysical and photochemical properties of RSP. **a** the absorption spectra in different buffers and DMF; **b** the reflectance spectra with the excitation wavelength of 610 nm in buffer and DMF; **c** the ultraviolet-visible absorption spectra of RSP and DPBF mixtures after laser irradiation for different lengths of time; **d** the absorption spectra after laser irradiation (225 mW/cm^2^)

Due to its amphiphilic nature, RSP had limited solubility in pure water, while it had good solubility in aqueous solutions with 1 % Cremophor EL (CEL). Therefore, we dissolved RSP in physiological saline containing 1% CEL, DMEM medium containing 0.05% CEL, and DMF (as a control) to measure its absorption and fluorescence spectrum. From the absorption spectra (Fig. 2a), it could be seen that RSP remained monomer in the physiological saline containing 1% CEL and DMEM medium containing 0.05% CEL, almost completely overlapped with the absorption spectrum in DMF, and the Q band absorption peak was sharp. As shown in Fig. 2b, the fluorescence intensity of RSP in physiological saline was higher than that in the organic solvent DMF, while the fluorescence intensity in DMEM is similar to that in DMF. RSP had the characteristics of not being aggregated in physiological saline and cell culture media, which provides a basis for further investigation of its phototoxicity.

In order to better simulate the situation of photosensitive molecules in practical applications, the singlet oxygen yield of RSP in physiological saline containing 1% CEL was measured (Fig. 2c). The slope of RSP and SC was 0.06481 and 0.07165 respectively (Supplementary Fig. 6). It could be seen that the introduction of cRGDyK did not significantly affect the singlet oxygen yield of photosensitive molecules, with only 10% decrease due to the steric hindrance, which may have a slight effect on the reaction between the oxygen and the phthalocyanine (Supplementary Fig. 7). We further determined the photostability of RSP. It could be seen that after 4 mins of irradiation, the absorbance at the maximum absorption wavelength of RSP only decreased slightly, remained about 80% of the original (Fig. 2d). The axial modification of RSP helped to improve the stability of the phthalocyanine parent ring when singlet oxygen was generated, so that it was less prone to degrade. The results of the investigation of light stability show that RSP has excellent photostability and could be suitable for long-term storage.

### Cellular uptake and subcellular co-localization

**Fig. 3:**
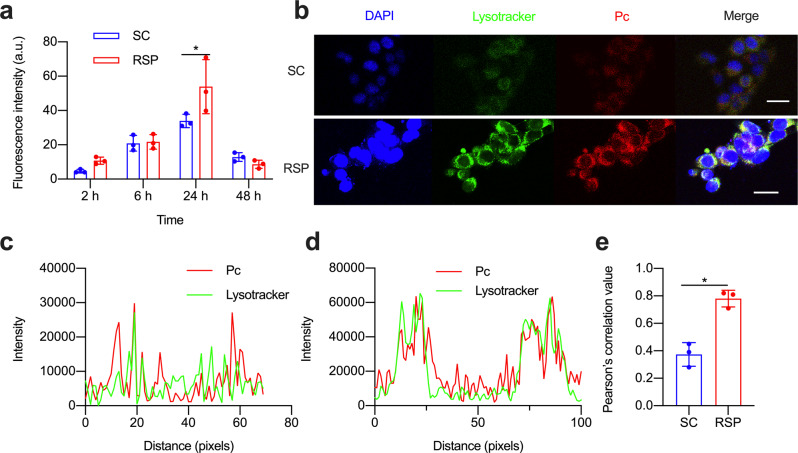
Cellular uptake and subcellular co-localization of RSP on 4T1 cells. **a** The quantitative analysis of the fluorescence changes of SC and RSP in 4T1 cells at different incubation times (*n* = 3); **b** Confocal imaging of SC and RSP co-localized in 4T1 cells (blue is the nucleus, green is the lysosome, and red is the fluorescence of phthalocyanine, the scale is 25 μm); Analysis of the co-localization of SC (**c**) and RSP (**d**) with lysosomes in 4T1 cell; **e** The results of Pearson co-localization of SC and RSP with lysosomes in 4T1 cells are summarized, blue indicates RSP, red indicates SC (*n* = 3, *p < 0.05)

To assess cellular uptake of RSP, we incubated RSP and SC (untargeted control) with triple-negative breast (4T1) cancer cells (Supplementary Fig. 8). We observed a time-dependent uptake in cancer cell lines 4T1, reaching a maximum after 24 h, and the intensity of RSP was 2-flods higher than that of SC (Fig. 3a), indicating that cRGDyK can effectively increase the cellular uptake of PSs. After incubation of RSP for 4 h, confocal imaging was performed to map the distribution of RSP and SC inside 4T1 cancer cells (Fig. 3b). The distribution positions of SC and RSP roughly overlap with lysosomes. The further analysis of co-localization can be realized by plot profile (Fig. 3c, d). The lysosomal colocalization index of RSP (0.78) was better than that of SC (0.38) by analyzing the Pearson co-localization coefficient of the whole image (Fig. 3e), which indicated that RSP can better localize to the lysosome.

### In vitro pharmacokinetics and photodynamic therapy

**Fig. 4:**
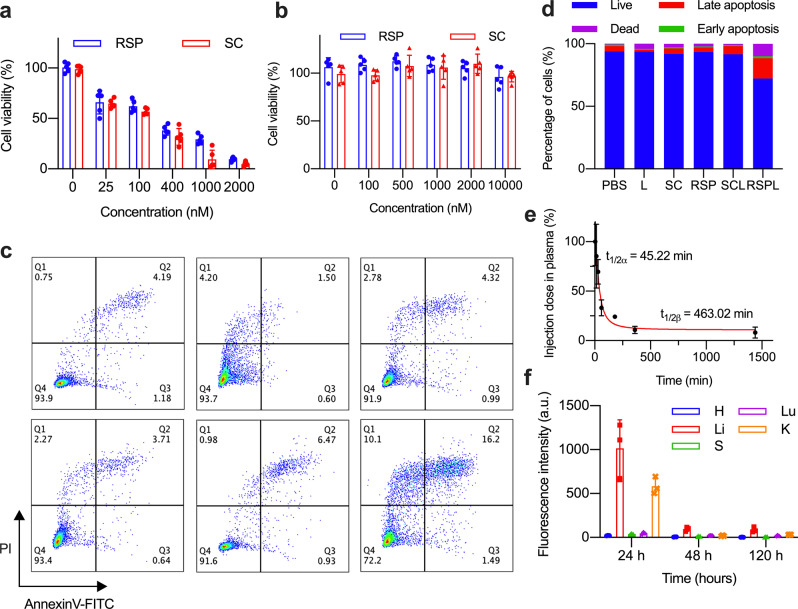
In vitro PDT of SC and RSP on 4T1 cells. (**a**) Dark toxicity and (**b**) phototoxic. (λ > 610 nm, 48 J/cm^2^, *n* = 5, *p < 0.05). **c** Analysis of the proportion of apoptosis and necrosis of cells after different treatments. **d** Quantification of the proportion of cells in different periods after different treatments. **e** The plasma drug metabolism curve of RSP. **f** The drug distribution in the main organs after intravenous injection of RSP (2 mg/kg) at different time points (*n* = 3, h is the heart, li is the liver, s is the spleen, lu is the lung, and k is the kidney)

The cancer-killing ability of RSP was examined by incubating 4T1 cancer cells with different concentrations for comparison with five groups: PBS only, light only (L), SC, RSP, SC + light (SCL) and RSP + light (RSPL). As shown in Fig. 4a, the cell viabilities of 4T1 cancer cells did no significantly decreased without light. However, the cell-killing capability of RSP and SC with light showed a prominent concentration-dependent ranging from 0 to 2–μM (Fig. 4b). Compared with the SC group, the RSP group exhibited a lower killing effect upon exposure to light (λ > 610 nm, 48 J/cm^2^), which was consistent with the singlet oxygen yield. Then, apoptotic, necrotic and live 4T1 cell populations were quantified by flow cytometric after TPDT (Fig. 4c, d). The apoptotic rate of cells incubated with RSP was increased by 9.73% compared with SC. All these experimental results indicated that RSP-based TPDT can effectively improve the therapeutic effect of PDT.

The ideal targeted photosensitizer should be able to quickly accumulate at the tumor site and metabolize from normal organs after injection. The investigation of the pharmacokinetics of RSP showed that plasma half-time was 45.22 min and elimination half-time was 463.02 min (Fig. 4e). After 24 h, 48 h, and 120 h of RSP injection, the major organs of the mice were dissected and their fluorescence was quantified. We found that RSP was mainly metabolized out of the body through the liver and kidney. After 120 h of injection, RSP had been completely metabolized out of the body, and no obvious fluorescent signal can be detected. These results indicated that RSP has a fast plasma clearance rate, and more than 90% RSP can be metabolized in 48 h. Long-term observation also showed that RSP has no tendency to accumulate specifically in organ (Fig. 4f), implying that RSP doesn’t have potential phototoxicity.

### In vivo tumor targeting and photodynamic therapy

**Fig. 5:**
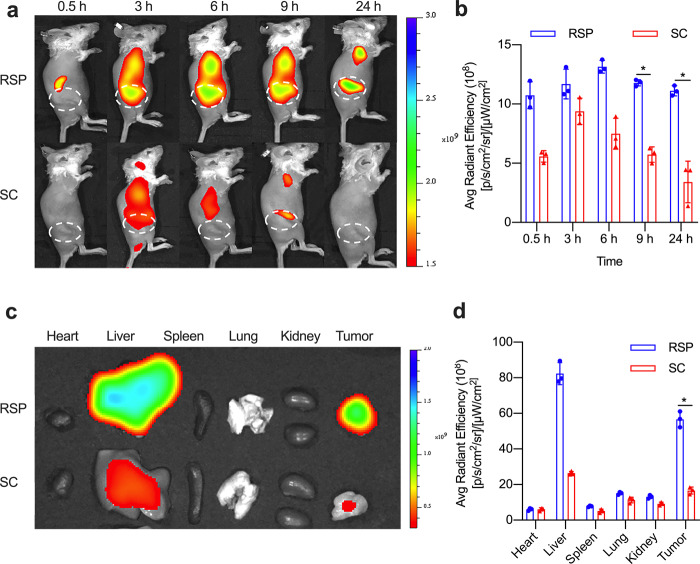
In vivo tumor targeting of RSP. **a** In vivo fluorescence imaging of the subcutaneous tumor model after injection of RSP and SC (2 mg/kg) at different time points. The dotted circle shows the tumor site. **b** The quantitative analysis of the fluorescence signal of the tumor site (*n* = 3). **c** In vitro fluorescence analysis of the main organs and tumors after 24 h injection. **d** The quantitative analysis of the main organs and tumors (*n* = 3, *P < 0.05, **P < 0.01)

The orthotopic and subcutaneous mouse model of triple-negative breast cancer were established and tumor imaging assay was conducted to investigated the in vivo tumor targeting of RSP. In the subcutaneous tumor model, the fluorescence signals for RSP and SC gradually increased with time after tail vein injections (Fig. 5a, b). For RSP, tumor accumulation was shown within 0.5 h, then, the signals continued to increase and reached peak intensity at 6 h after injection. Although the fluorescence intensity gradually decreased after 6 h, a strong signal was still observed until 24 h. We found that the tumor targeting of RSP was significantly better than that of SC. After 24 h of injection, the fluorescence intensity of RSP tumor sites was 2.5 folds higher than that of SC. To investigate the deposition pathway in organs, the organs were dismembered and inspected after 24 h injection in fluorescence imaging system with or without targeted modifications (Fig. 5c, d). Obviously, there is a significant difference in the tumor distribution of RSP and SC. The semiquantitative analysis showed that the fluorescence signal of RSP was four folds higher than that of SC at the tumor site. It was also found that more RSP was accumulated in the orthotopic tumor model than SC (Supplementary Fig. 9). The results of in vivo tumor imaging show that RSP has good tumor targeting and can remain at the neoplastic up to 24 h.

**Fig. 6:**
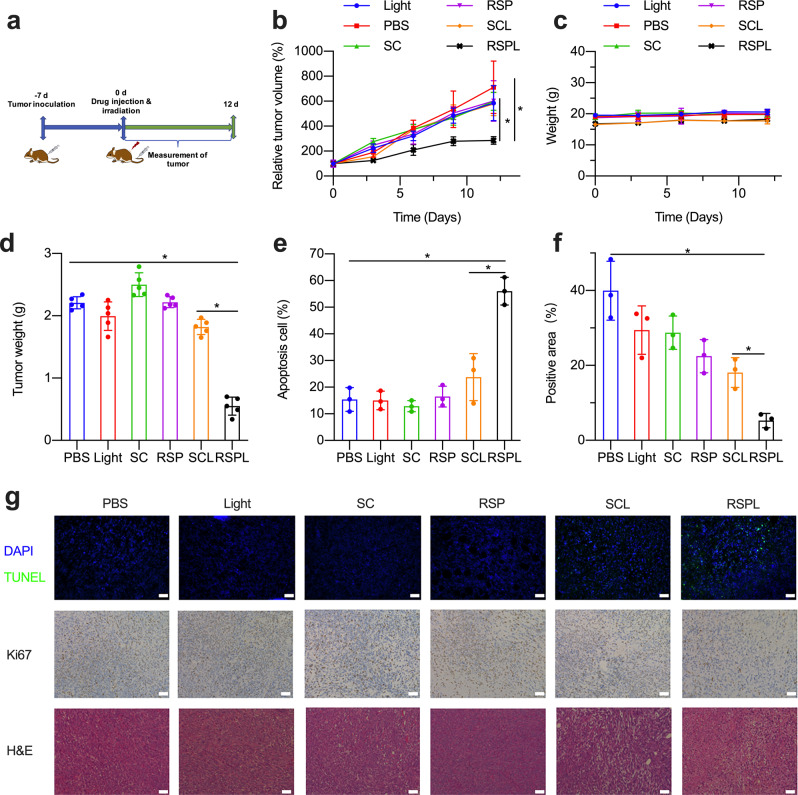
In vivo photodynamic therapy and biological safety of RSP. **a** The schematic diagram of RSP for tumor PDT; **b** The curve of mouse tumor volume after different treatments (*n* = 5, *p < 0.05); **c** Body weight of mice in different treatment groups (*n* = 5); **d** The tumor weight statistics of mice in different treatment groups at the end of PDT (*n* = 5, *p < 0.05). **e** Proportion of apoptotic cells in tumor tissue (*n* = 3, *p < 0.05). **f** Quantitative analysis of Ki67 immunohistochemical results in tumor tissue sections (*n* = 3, *p < 0.05). **g** TUNEL, H&E, and Ki67 immunohistochemical staining of tumor sections of mice in different treatment groups (The scale is 10 μm)

The encouraging fluorescence imaging results thrilled us to further investigate the in vivo photodynamic efficacy of RSP in 4T1-tumor bearing mice. As shown in Fig. 6c, the tumors in the RSPL group were effectively suppressed and their tumor volume was only 35.7% of that in the control group. There was no significant difference between the PBS, Light, SC, and RSP group in tumor volume. With regard to mouse body weight, we found no significant changes in all groups (Fig. 6c). At the end of the treatment, the tumors of the mice in each group were weighed. From Fig. 6d, the tumor suppression rate in the RSPL group reached 74% compared with the PBS group, while the proliferation of tumor cells in the SC-light group was not significantly inhibited.

By H&E staining and TUNEL staining analysis of the tumor site, the strong green fluorescent signal of tumor cell sections in the RSPL group indicates a high rate of apoptosis of cancer cells, which accelerates the inhibition of tumor growth. The SCL (SC + Light) group showed only a slight green fluorescence demonstrating that the RSP targeting in vivo was much better than SC. After quantitative analysis of the fluorescence signals of the tissue, it was found that the apoptosis in the RSPL group was twice that of SCL group (Fig. 6e). These results suggested that TPDT could induce additional apoptosis. Ki67 immunohistochemical staining could reveal that the RSPL group could significantly inhibit tumor growth, while the other groups were brown positive markers in the whole field and tumor cells proliferated very vigorously. From the quantitative analysis results, it can be seen that the Ki67 proliferation signal of the RSPL group is only 10% of that of the PBS control group, which is 1/4 of that of the SCL group (Fig. 6f), which also strongly illustrates that TPDT can more fully inhibit the proliferation of tumor cells than nontargeted PDT. In order to better evaluate the safety of TPDT, the main organs of mice in different treatment groups were taken out and H&E stained for pathological analysis (Supplementary Fig. 10). It can be found that the H&E sections of the heart, liver, spleen, lung, and kidney have no obvious pathological phenomena. This shows that the injection of RSP and laser irradiation during the treatment process did not produce toxic side effects on the body, which further proves that the use of RSP for TPDT is safe.

## Discussion

As photodynamic therapy progresses toward clinical translation, more precise photosensitizer enrichment becomes mandatory. Targeted modification of PS to increase the peak concentration of photosensitizer at the tumor site is the current focus of research. The predominant approach for targeted modification of small molecule drugs is antibody-drug conjugate (ADC).^33,34^ ADC can precisely deliver drugs to tumor cells through highly specific antibody-receptor-specific recognition.^35^ While enhancing the uptake by tumor cells, it reduces the nonspecific uptake of photosensitizers by normal cells. In addition, smaller-sized nano-antibodies have been shown to improve the targeting ability of drugs. However, the large size of antibodies limits their penetration into tumors, and the high cost of production limits their use in a few clinical sites. In contrast, modification of drugs using small molecule peptides with high tumor specificity can provide comparable targeting ability to antibody drugs while reducing the production cost. We synthesized amphiphilic and asymmetric targeted silicone phthalocyanines using targeted peptides. Its smaller molecular size has better penetration ability. The cost of targeted modifications has been reduced remarkably, making it more suitable for large-scale synthesis. Therefore, RSP has better prospects for clinical translation than ADC.

The cRGDyK peptide is the most widely studied class of tumor-specific peptides. There have been many preclinical experiments showing that the use of cRGDyK coupled with imaging agents (microbubbles, magnetic resonance contrast agents, fluorescein, etc.)^36–38^ can effectively improve the sensitivity of contrast agents for tumor imaging, which confirms the broad tumor-targeting effect of cRGDyK. In addition, RGD has been widely used to improve the targeting of small molecule photosensitizers or nano-photosensitizers. Previous studies have reported overexpression of integrins in some tumors, including triple-negative breast cancer, which have led to the development molecular probes for the treatment of the RGD base.^39–42^ Although these studies modified phthalocyanine with cRGDyK peptide, they still failed to achieve successful clinical translation. The most important reasons for this are poor water solubility and complex synthesis routes. Poor water solubility leads to aggregation in aqueous solutions of phthalocyanines with reduced single-linear oxygen yields. To increase water solubility, drug excipients that promote solubility or inhibit aggregation of photosensitizers are added, which adds a lot of unnecessary burden to metabolism. Considering these problems, we modified the phthalocyanines axially rather than on the rings. The formation of axial chemical bonds hinders the interaction between the phthalocyanine rings, which effectively avoids the aggregation of photosensitizers. Moreover, RGD was introduced not only as a targeting group but also as a hydrophilic group. The increase of water solubility further reduces the aggregation of phthalocyanine. cRGDyK molecule enhances both the targeting ability and hydrophilicity of RSP, which effectively solves the problem of targeting phthalocyanine aggregation.

In our further study, we found that after axial modification of RGD, phthalocyanine not only increased targeting but also significantly reduced aggregation in aqueous solution. In previous studies, the fluorescence intensity of silicone phthalocyanine in aqueous solution was significantly lower than that in organic solvents.^43^ The fluorescence spectroscopy experiments showed that RSP buffer under physiological conditions had higher fluorescence intensity than in DMF. Meanwhile, the Q-band absorption peak of RSP in physiological saline containing 1% CEL was sharp. These results indicated that RSP is in the form of monomer in physiological buffers. The axial modification of phthalocyanine effectively avoided the formation of H-aggregates and J-aggregates. Further experiments also demonstrated that RSP exhibited good singlet oxygen yield and photostability in physiological buffers. The modification of RGD resulted in a high accumulation of photosensitizer in triple-negative breast cancer cells. In vitro photodynamic experiments showed that the IC50 of RSP in 4T1 cell line was 295.96 nM, which could cause significant apoptosis of tumor cells. And RSP showed high tumor suppression rate for triple-negative breast cancer tumors in orthotopic mice in vivo. In addition, RSP has a circulating half-life of approximately 45 min and is metabolized by the liver and kidney. In conclusion, RSP showed superior tumor targeting than non-targeted silicone phthalocyanine and could inhibit tumor growth more effectively.

RSP showed great promise for improving the photodynamic therapeutic efficacy of triple-negative breast cancer versus the untargeted photosensitizer. Both in vivo and in vitro, the targeted photosensitizer RSP exhibited better comparative targeting and tumor suppression in triple-negative breast cancer, as compared to the non-targeted silicone phthalocyanine. Although monoclonal antibodies remain the mainstay of targeted therapies, their large size limits their penetration into tumors, and high production costs limit their usage in a few clinical centers. However small molecule agents like RSP, which can be synthesized inexpensively on a large scale, represents a promising next-generation PDT modality. Our work addresses current research needs and most definitely study will help expand the currently limited range of PDT cancer therapies available. Based on RSP, silicone phthalocyanine can not only be conjugate to cRGDyK, but also can be replaced with different peptides according to different tumor types to achieve photodynamic therapy for different kinds of tumors. Further it can be connected to different imaging contrast agents, such as MRI contrast agent, SPECT contrast agent, to achieve impact-guided photodynamic therapy, making it a multifunctional molecule for theranostics.

In summary, we demonstrated that covalent linkage of RGD in RSP can effectively enhance the uptake of integrin-expressing tumor cells, hence improving the efficiency of PDT. In physiological buffers, this photosensitizer RSP has excellent fluorescence properties and singlet oxygen production, and exhibits high stability under light. Both in vivo and in vitro, the targeted photosensitizer RSP exhibited better comparative targeting and tumor suppression in triple-negative breast cancer, as compared to the nontargeted silicone phthalocyanine. Consequently, the targeted silicon phthalocyanine synthesized in this paper has a simple synthesis route, strong targeting, high photosensitivity, and has a strong prospect for transformation and application. There are ongoing efforts to evaluate the efficacy of our small molecule probes for clinical imaging and therapy. We expect that the successful development of these agents will expand the currently limited range of cancer therapies available.

## Materials and methods

### Instrumentation and materials

Silicon phthalocyanine dichloride was synthesized according to the reported method.^44^ All reagents, including 3-(4-Hydroxyphenyl)propionic acid, sodium hydride, EDC·HCl, NHS, cyclo(Arg-Gly-Asp-d-Tyr-Lys) (cRGDyK), diethyl ether and dichloromethane are commercially available and were used without further purification. Nuclear magnetic resonance (NMR) spectra were recorded on a Brüker Avance 500 spectrometer at 25 °C. Chemical shifts were reported in parts per million (ppm) downfield from the Me4Si resonance which was used as the internal standard when recording ^1^H NMR spectra. Mass spectra (MS) were obtained on Solarix XR. UV–vis absorption spectra were obtained on Thermo Fisher UV–vis spectrophotometer. Fluorescence spectra of the different samples were obtained on Thermo Fisher fluorescence spectrophotometer. HPLC assays were performed on a C18 column (5 mm, 4.6 mm 250 mm). solvent A: 100% H_2_O containing 0.1% TFA; solvent B: 100% DMF containing 0.1% TFA. The column temperature was 30 °C and the flow rate was 1.0 ml/min. The elution gradient was: 10-95% B for 20 min, then 95% B for 20 min.

### Synthesis of 2[4-(3-carboxypropyl)phenoxy]silicon phthalocyanine (SC)

Silicon phthalocyanine dichloride (100 mg), 3-(4-Hydroxyphenyl)propionic acid (108 mg), and sodium hydride (16.4 mg) were added to a 100 mL round bottom flask, dissolved in 15 mL anhydrous toluene, and heated to reflux for 24 h. Then the mixture was cooled to room temperature. The solvent was spin-dried, and the obtained solid was washed with water and filtered. The obtained residue was fully dissolved in DMF and filtered. The filtrate was spin-dried and dried to obtain 2[4-(3-carboxypropyl)phenoxy]silicon phthalocyanine (SC)(57 mg, 39% yield). HRMS (ESI): m/z calcd for C_50_H_35_N_8_O_6_Si [M + H] + , 871.2443; found 871.2426.

### Synthesis of cRGDyK modified targeting silicon phthalocyanine (RSP)

SC (20 mg), EDC·HCl (4.4 mg) and NHS (2.6 mg) obtained in the previous step were dissolved in 2 mL of anhydrous DMF, stirred at room temperature for 4 h, and then cRGDyK (4.27 mg) and the solution were added to the mixture and stirred overnight at room temperature. The mixture was poured into excess diethyl ether and filtered. After the filter residue was collected and fully washed by water after being fully washed with dichloromethane. The resulting precipitate was vacuum dried to obtain cRGDyK modified targeted silicon phthalocyanine (RSP) (2.43 mg, 24% yield). (^1^H NMR (500 MHz, DMSO) δ 9.67 (ddd, J = 8.4,7.8, 4.3 Hz, 8H), 9.15 (s, 1H),8.86 (s, 1H), 8.54 (dt, J = 7.9, 6.7 Hz, 8H), 8.29 (s,2H), 7.93 (s, 1H), 7.76 (m, 5H),7.20-6.99 (m, 6H), 6.64 (dd, J = 8.4, 3.0 Hz, 2H), 6.42 (dd, J = 8.5, 2.1 Hz, 2H), 5.97 (m, 1H), 5.41 (d, J = 7.5 Hz, 3H),5.14 (m, 1H), 4.50 (s, 2H), 4.08 – 3.93 (m, 2H), 3.21-2.90 (m,4H), 2.76-2.58 (m, 8H), 2.34-2.09(m, 4H), 1.84 (s, 4H), 1.47 (s, 4H), 1.04(m, 2H).) HRMS (ESI): m/z calcd for C_77_H_74_N_17_O_13_Si [M + H] + , 1472.5443; found 1472.5406. HPLC (680 nm): >96%.

### Singlet oxygen yield detection

Generation of singlet oxygen was detected chemically using the disodium salt of 1,3-diphenylisobenzofuran (DPBF) as a singlet oxygen sensor. DPBF is bleached by singlet oxygen to its corresponding endoperoxide. The reaction was monitored spectrophotometrically by recording the decrease in optical density at 414 nm. The bleaching rate of DPBF can also be used as a method to compare the yield of singlet oxygen. In the actual measurement of singlet oxygen yield, a certain amount of DPBF ethanol solution and test solution are mixed together in a dark room. A rotor is added to the cuvette, and the laser is irradiated for different times while stirring. The absorption spectra of the mixed solution was tested after different time of irradiation with an ultraviolet spectrophotometer, then the change of the absorption value of DPBF at 414 nm was obtained.

### Cell culture studies with tumor cells

Luciferase labeled murine triple-negative breast cancer (4T1-Luc2 ATCC® CRL-2539-LUC2) cells were routinely cultured in DMEM medium (GIBCO) containing 10% fetal bovine serum (FBS). All experiments were performed at room temperature unless otherwise stated. CEL was obtained from Sigma. CCK8 cell activity assay kits were from Beyoncé. DCFHDA reactive oxygen species assay kits were purchased from KGI. Lysosomal probe, DAPI, and apoptosis assay kits were purchased from Solebro.

### Cell uptake and imaging

4T1 cells were seeded and cultured overnight at a density of 10,000 cells/well. On the second day, the medium was changed to DMEM medium containing 1 μM RSP or SC (containing 0.05% CEL) or DMEM medium. Three cells were set up in duplicate wells at each observation time point. At different time points after incubation, the cells were taken out of the incubator, the medium containing the photosensitizer was discarded, and the cells were incubated with a fresh medium. The fluorescence inside the cells was observed under a fluorescence microscope and photographed at least three fields at each time point. Image J was used for quantitative analysis.

Subcellular colocalization pictures were taken under a laser confocal microscope (Nikon A1R high-speed confocal laser microscope, Nikon Corporation, Japan). 4T1 cells were cultured in 35 mm cell culture dishes at a density of 20,000 cells/dish, and cultured overnight. After 24 h, the medium was replaced with DMEM medium (containing 0.05% CEL) containing 1 μM RSP or SC. After 4 h of incubation, the plates were washed with PBS and then stained with DMEM medium containing Lysotracker for 30 min and washed again with PBS. Then fixed and counterstained with DAPI. The light was always avoided during the entire operation. The dynamic change of fluorescence on any straight line passing through the whole cell was analyzed by Image J. The result was plot to qualitatively analyze the co-localization situation of lysosome and phthalocyanine. In order to more completely reflect the co-localization of lysosomes and phthalocyanines, the Pearson co-localization coefficient was analyzed on the fluorescence fields of cells obtained from three photographs, and the difference in lysosomal localization of RSP and SC in the cells was quantitatively analyzed.

### Intracellular singlet oxygen detection

DCFHDA, a small molecule probe that can freely cross the cell membrane, was used as the probe to detect intracellular singlet oxygen. The generation of singlet oxygen can be judged by whether or not it produces green fluorescence. 4T1 cells were seeded in a 96-well plate at a density of 10,000 cells/well. After 24 h, the medium was replaced with DMEM medium containing 1 μM RSP or SC (containing 0.05% CEL). The cells were incubated for 4 h. Then the cells were taken out of the incubator, add DMEM medium containing 10 μM DCFHDA, and continue incubating for 30 min. After the incubation, the original medium was discarded, replaced with a fresh DMEM medium, and irradiated with a xenon lamp. After irradiation for different periods of time, it was detected with Enzyme Labels to determine the production of reactive oxygen species in the cells. Then observe the green fluorescence generated in the cells under a fluorescence microscope.

### Cytotoxicity assay

4T1 cells were seeded in a 96-well plate at a density of 10,000 cells/well, and each well was cultured overnight with 200 μL of DMEM medium containing 10% FBS. On the second day, the original medium was discarded and replaced with DMEM medium containing different concentrations of SC or RSP (containing 0.05% CEL). After 4 h of incubation, the cells were incubated with fresh medium and irradiated with a xenon lamp. Then 10 μL of CCK8 was added to each well, and the incubation continued for 1 h. After the incubation, the absorbance value at 450 nm was measured with enzyme labels (Synergy HT, BioTek, USA). At the same time, a blank control was set to determine cell viability. The dark toxicity test method was similar, except that no light was applied to the cells.

The apoptosis rate and necrosis rate of the cells were detected on the flow cytometer according to the operating procedure of the apoptosis detection kit. 4T1 cells were seeded in a six-well plate at a density of 5 * 10^5^ cells/well overnight. On the second day, the medium was changed to DMEM medium containing 500 nM RSP/SC and 1% CEL, and the cells were incubated for 4 h. Then the medium was replaced with fresh DMEM. The cells were applied light/ no light, and then digested.

### Animal experiments

Balb/c female mice aged 6 to 8 weeks were purchased from Beijing Weitong Lihua Laboratory Animal Technology. All animal experiments were performed under protocols approved by the Institutional Animal Care and Use Committee (IACUC) of Peking University. In situ breast cancer tumor models were established by injecting 2 * 10^7^ 4T1-luci tumor cells under the fourth pair of mammary glands in mice. Treatment was initiated approximately 6 days after inoculation when the tumor volume grew to approximately 100 mm^3^.

### Blood circulation dynamics investigation

In order to investigate the blood clearance rate of RSP, healthy male Balb/c mice were selected as experimental subjects, and blood was collected from the orbit to detect the drug concentration in the body at different time points. Fluorescence labeled Organism Bioimaging Instrument (FOBI, South Korea) was used to measure the relative concentration of phthalocyanine in mouse plasma. Mice were injected with RSP through the tail vein at a dose of 2 mg/kg. At different time intervals, blood samples were collected from the orbits of mice with capillary tubes coated with heparin, placed on ice, and centrifugated to separate blood cells (3000 g, 10 min). The resulting plasma samples were analyzed for phthalocyanine concentration.

### In vivo fluorescence imaging

For in vivo fluorescence imaging, RSP or SC dissolved in physiological saline (containing 1% CEL) was injected intravenously at a dose of 2 mg/kg into tumor-bearing mice (both in situ and subcutaneous models). Then IVIS Spectrum In Vivo Imaging System (PerkinElmer) was utilized to collect the fluorescence signals of RSP (660 nm/710 nm) in subcutaneous models at different times after the injection. The small animal fluorescence imaging system was used for in situ tumor-bearing mice imaging, taking pictures, and recording. The excitation wavelength was 660 nm, and the fluorescence signal was collected with a 700 nm filter. Then, the FOBI software was used to process the data to analyze the enrichment of phthalocyanines in the tumors of the mice after different injection times. The mice after 24 h fluorescence imaging were sacrificed, and the hearts, livers, spleens, lungs, kidneys, and tumors were collected for analysis of fluorescence semiquantitative biodistribution.

### Evaluation of in vivo photodynamic activity

PDT treatment was performed on the 7th day when the 4T1 tumor model was established in mice, and the average initial tumor volume was 100 mm^3^. The test groups were as follows: Group 1: PBS; Group 2: Light; Group 3: SC; Group 4: RSP; Group 5: SC + Light, Group 6: RSP + Light. First, 200 μl of RSP (2 mg/kg), SC (2 mg/kg), and PBS were injected intravenously. 4 h after the injection, the tumor area was irradiated with 671 nm laser (0.1 W/cm^2^) for 6 min with a total laser intensity of 36 J/cm^2^ and laser treatment was performed on groups 2, 5, and 6. After PDT treatment, the tumor size was measured every day using electronic vernier calipers until 12 days. The maximum width (X) and length (Y) of the tumor were measured, and the following formula was used to calculate the tumor volume: *V* = (X^2^Y)/2. From the first day of treatment, the weight change of each group of mice was recorded until the end of the treatment. Mice in each group were selected randomly, and excised tumors for pathological analysis. In order to investigate the biological safety of this PDT method, after the treatment, the main organs (heart, liver, spleen, lung, kidney) of each group of differently treated mice were dissected, and H&E section staining was performed to evaluate their Pathological changes.

## Supplementary information

Supplementary information

## Data Availability

On reasonable request, the corresponding author will provide all data supporting the findings of this study.
