# Supplementary material for: Asymmetric, amphiphilic RGD conjugated phthalocyanine for targeted photodynamic therapy of triple negative breast cancer
Source: Signal Transduct Target Ther. 2022 Feb 28;7:64. doi: 10.1038/s41392-022-00906-2 (PMC8885659; doi:10.1038/s41392-022-00906-2)
Supplement: Supplementary file 1 — Supplementary information [file 41392_2022_906_MOESM1_ESM.docx]

Supplementary Materials for

Asymmetric, amphiphilic RGD conjugated phthalocyanine for targeted photodynamic therapy of triple negative breast cancer

Rui Li ^1#^, Yiming Zhou ^1#^, Yijia Liu ^1^，Xingpeng Jiang ^1^, Wenlong Zeng ^1^, Zhuoran Gong ^1^, Gang Zheng ^2,3^, Desheng Sun ^4*^, Zhifei Dai ^1*^

Correspondence to: Zhifei Dai (zhifei.dai@pku.edu.cn); Desheng Sun ([szdssun@163.com](mailto:szdssun@163.com)）

# These authors contributed equally to this work.

**This PDF file includes:**

Figures. S1 to S10


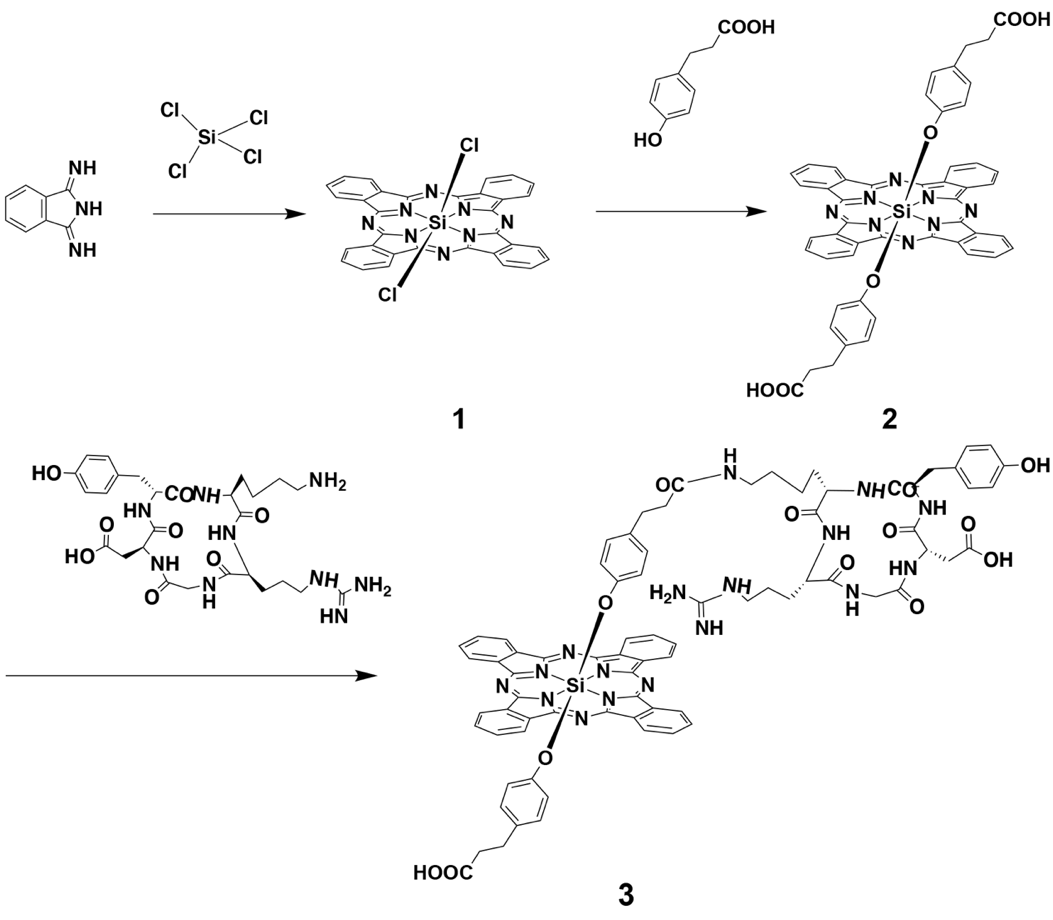


Figure. S1.

Synthetic route for RSP.


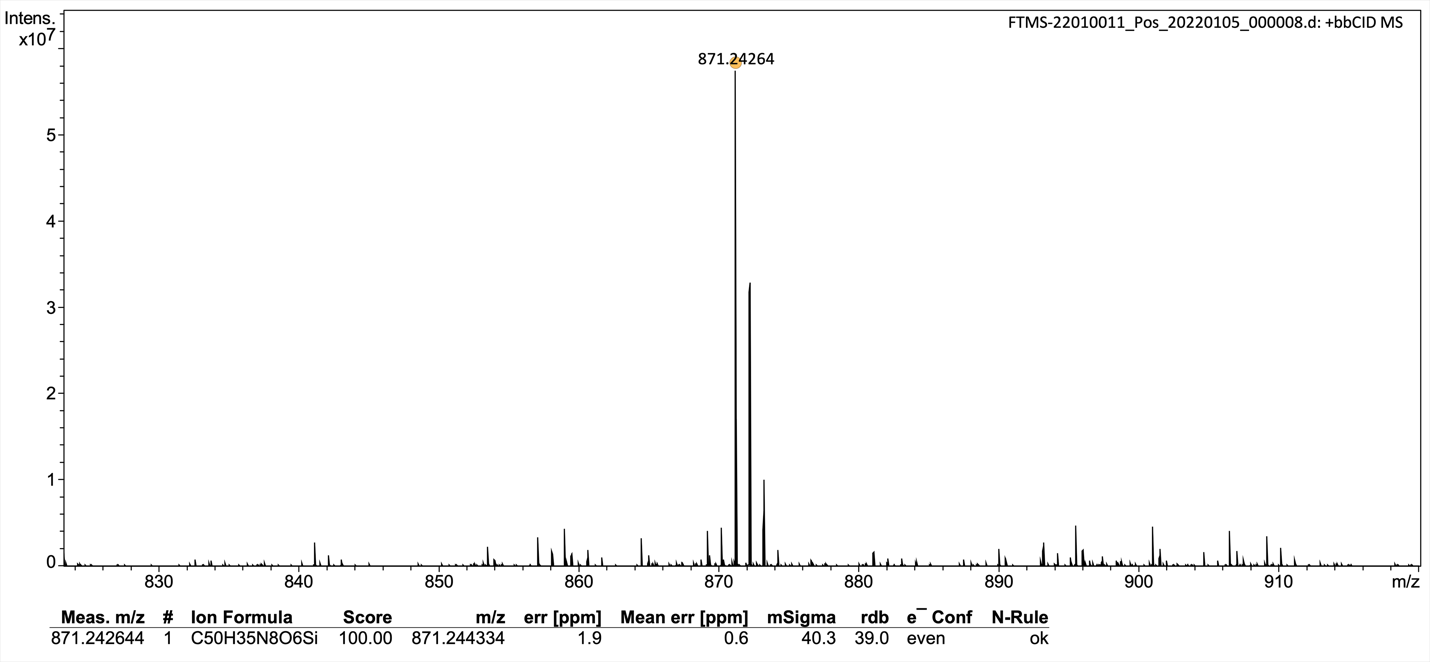


Figure. S2.

ESI-Mass spectra of SC.


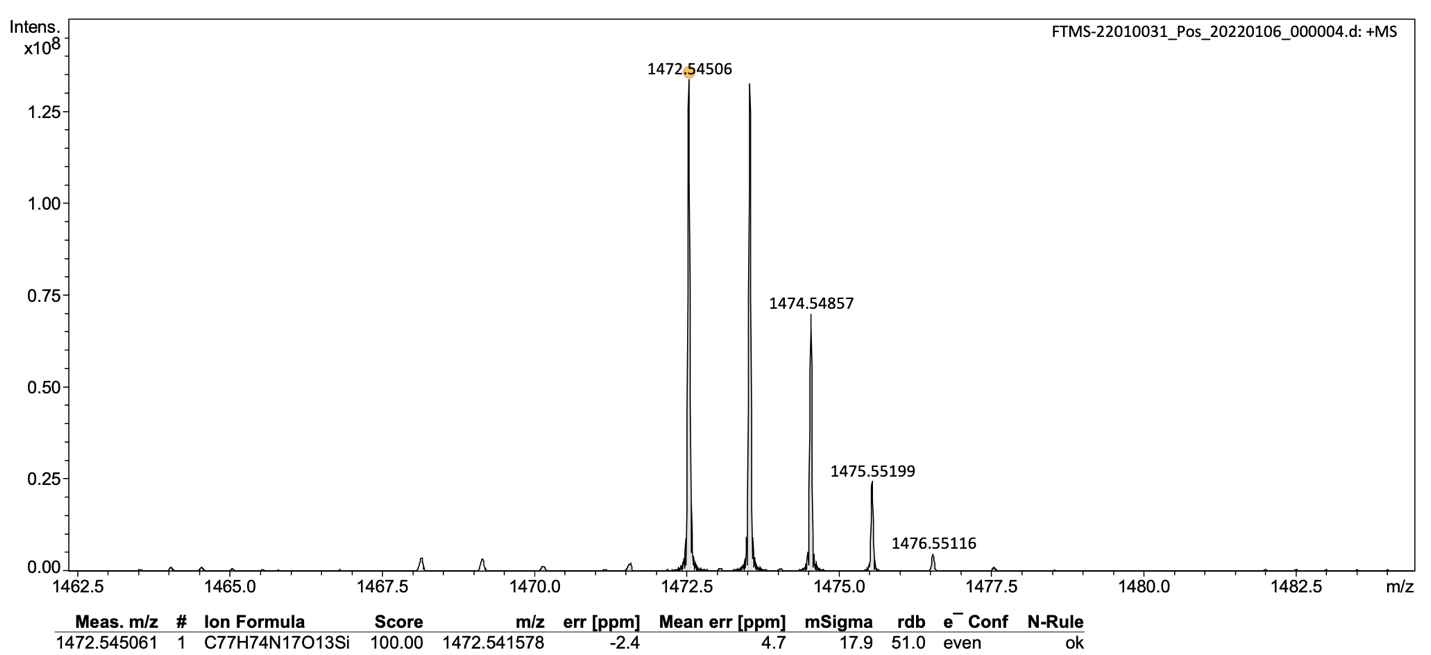


Figure. S3.

ESI-Mass spectra of RSP.


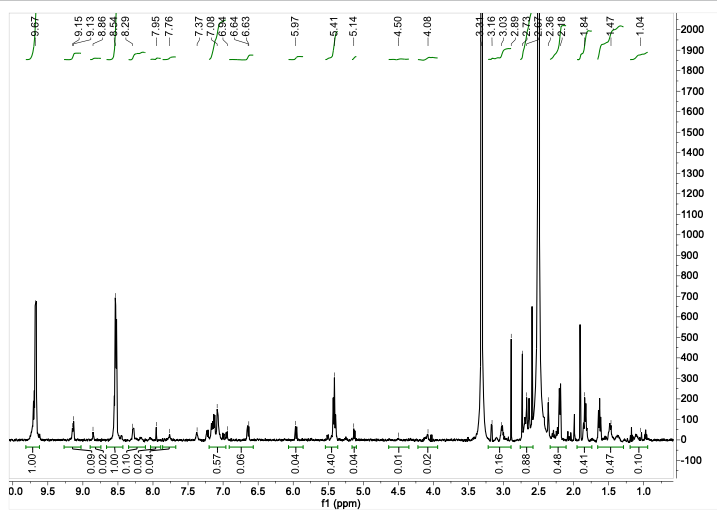


Figure. S4.

1H NMR spectrum of (500 MHz, DMSO, 25 ^o^C) of RSP.


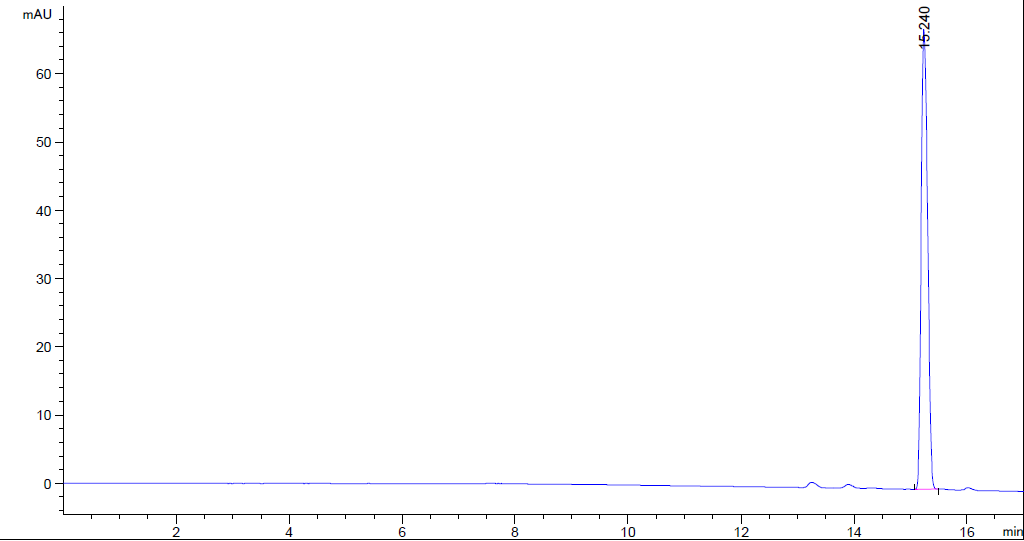


Figure. S5.

HPLC analysis of RSP (t_R_ = 15.24 min). Wavelength for detection: 680 nm.


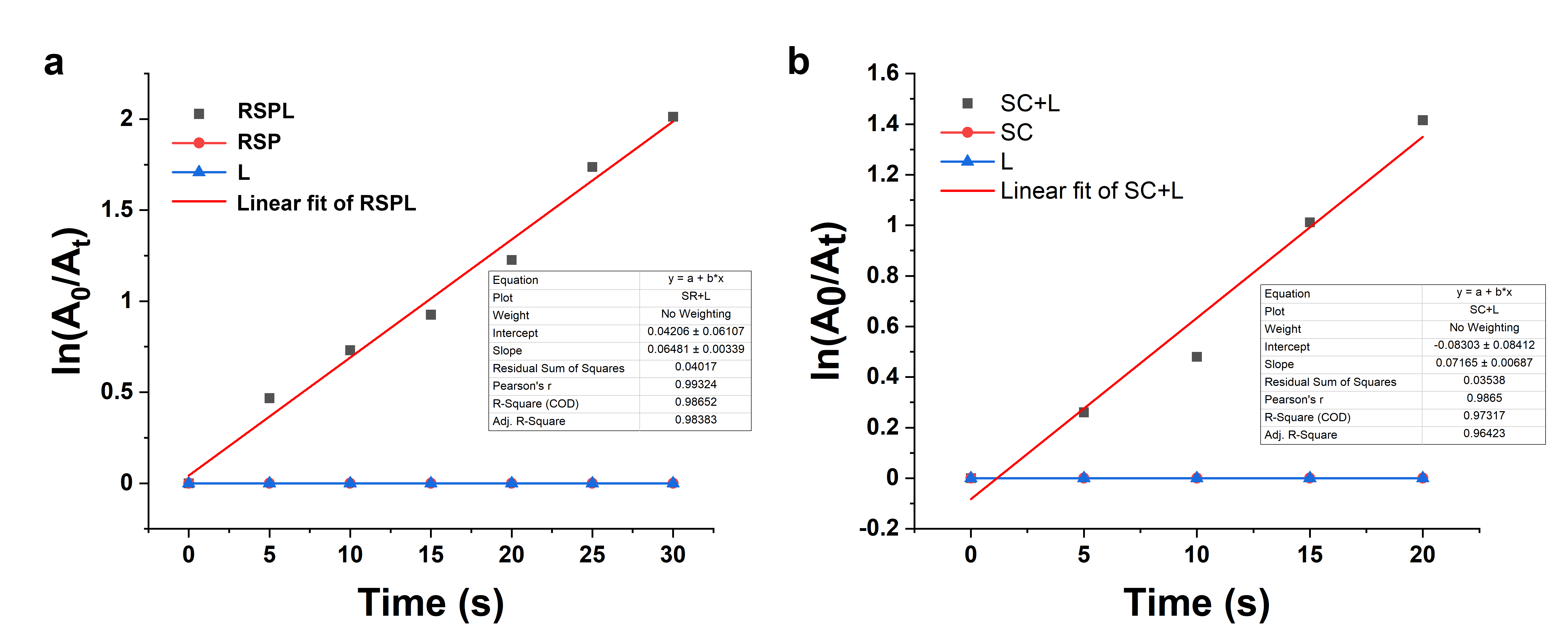


Figure. S6.

Singlet oxygen quantum yields of RSP and SC after laser irradiation (610 nm, 225 mW/cm^2^) in physiological saline containing 1% CEL.


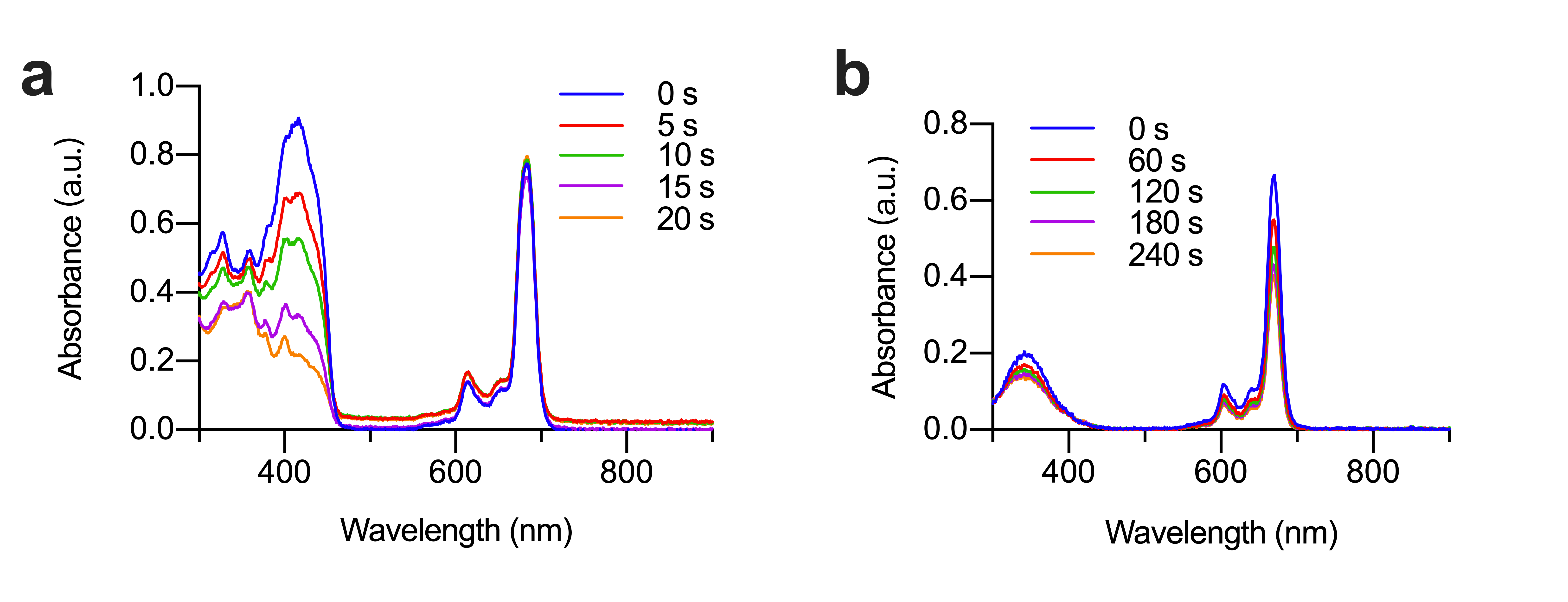


Figure. S7.

**a** Absorption spectra of SC and DPBF mixtures after laser irradiation for different time. **b** Absorption spectra of Zinc phthalocyanine after laser irradiation (610 nm, 225 mW/cm^2^).


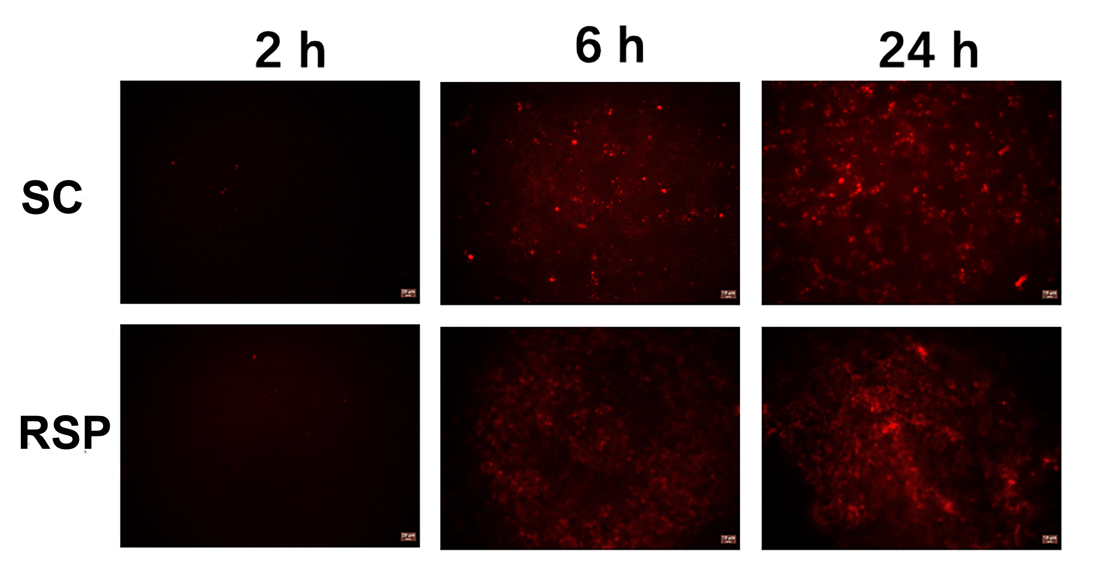


Figure. S8.

Fluorescence imagines of SC and RSP in 4T1 cells at different incubation times (the scale is 10 μm).


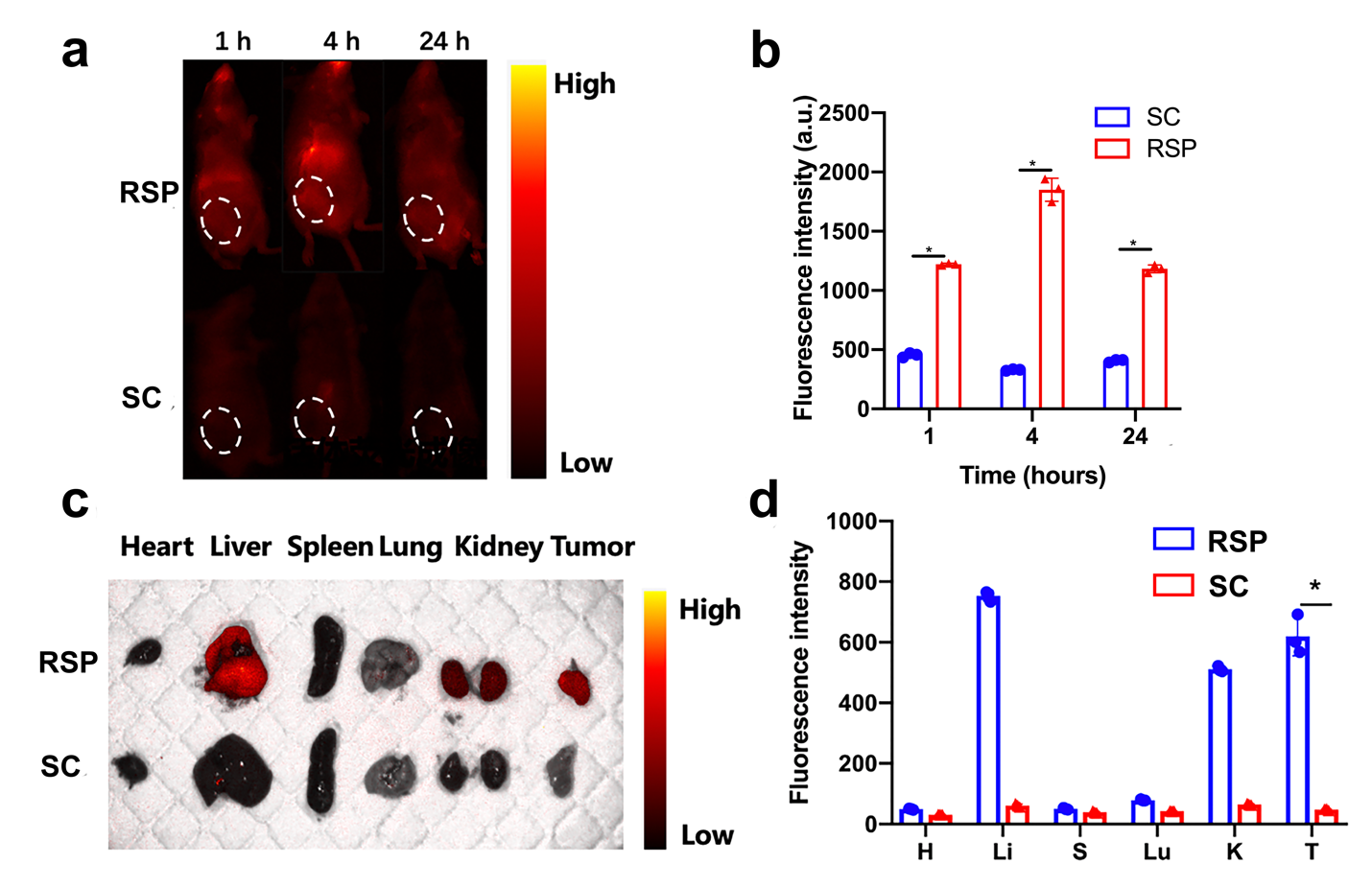
Figure. S9.

**a** *In vivo* fluorescence imaging of orthotopic breast cancer carrying mice after injection of RSP and SC (2 mg/kg) at different time points. The dotted circle shows the tumor site. **b** The quantitative analysis of the fluorescence signal of the tumor site (n = 3). **c** *In vitro* fluorescence analysis of the main organs and tumors. **d** The quantitative analysis of the main organs and tumors (n = 3, h is the heart, li is the liver, s is the spleen, lu is the lung, and k is the kidney).


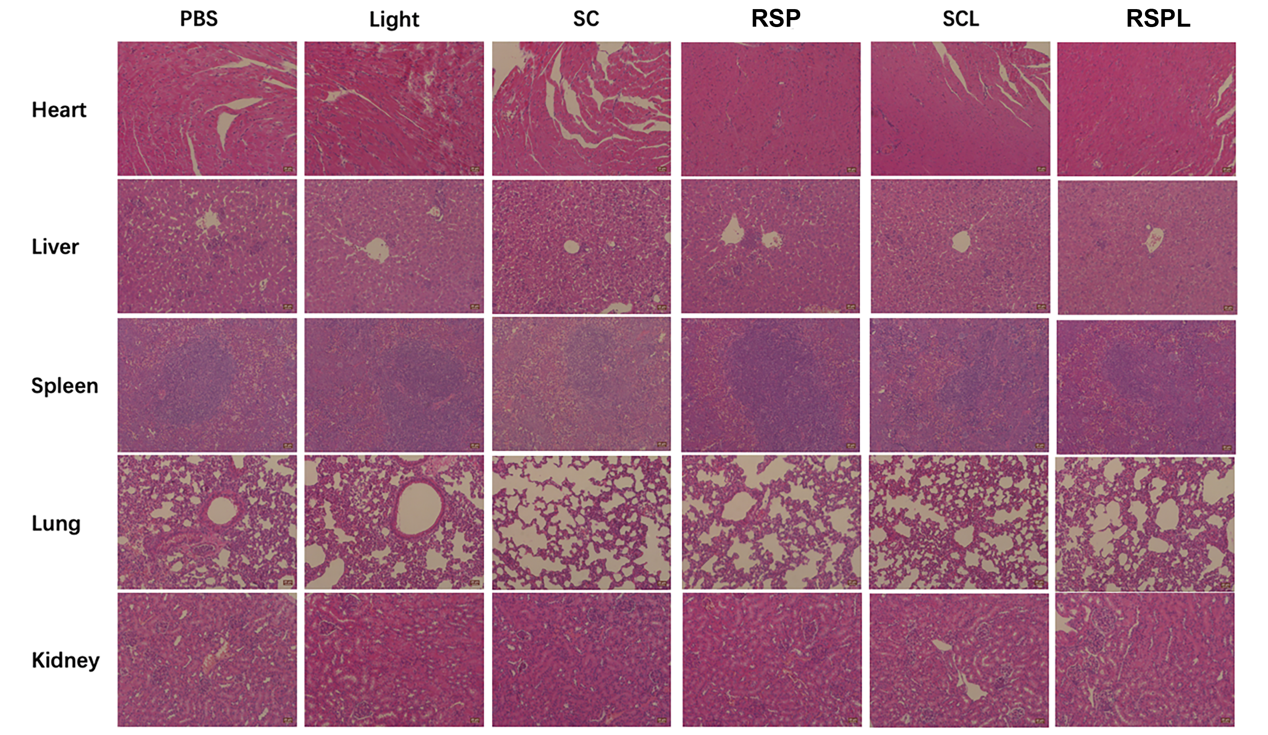


Figure. S10.

H&E staining of the main organs of mice in different treatment groups (the scale is 20 μm).
